# Circadian Variation of Salivary Oxytocin in Young Adult Women

**DOI:** 10.1111/psyp.70139

**Published:** 2025-09-01

**Authors:** M. Teixeira de Almeida, L. Quattrocchi, N. Perroud, T. Aboulafia‐Brakha

**Affiliations:** ^1^ Faculty of Medicine University of Geneva Geneva Switzerland; ^2^ Department of Psychiatry Geneva University Hospitals Geneva Switzerland

**Keywords:** borderline personality disorder, circadian variation, female participants, hormonal cycle, oxytocin, salivary biomarkers, young adult women

## Abstract

This article presents circadian variation in salivary oxytocin levels in a sample of 91 female participants, including 47 healthy controls and 44 patients with borderline personality disorder (BPD). A significant increase in salivary oxytocin levels was observed between awakening and early afternoon. There were no significant group differences and no Group × Time interaction. These findings have implications for research conducted in the field and suggest the need to control for time of assessment, as done in studies assessing cortisol.

## Abstract 2

This study provides evidence of circadian variation in salivary oxytocin levels in young adult women, observed in both healthy controls and participants with borderline personality disorder (BPD). These findings highlight the importance of considering time‐of‐day effects when studying oxytocin's role in psychological processes.

## 1 Introduction

The neuropeptide oxytocin is increasingly being studied for its role in social bonding, emotion regulation, stress response, and cognitive flexibility (Audunsdottir and Quintana 2022; Carter et al. 2024; Quintana and Guastella 2020; Yao and Kendrick 2025; Kirsch 2015). Endogenous oxytocin measures are usually collected under basal conditions or during experimental designs in both clinical samples and healthy individuals (Carter et al. 2024; Martins et al. 2020). An important question that has received relatively little attention is whether oxytocin displays circadian variation similar to the well‐established variations of cortisol (Van Dam et al. 2018; Ryan et al. 2016; Stalder et al. 2022). This question is particularly relevant for methodological aspects and could partially explain significant data variability and mixed findings in the field (Martins et al. 2020). However, assessing circadian variation requires at least two or more measurements throughout the day at specific time points, whereas quantification of oxytocin in blood or cerebrospinal fluid (CSF) may limit this type of assessment in large samples (Van Dam et al. 2018; Graugaard‐Jensen et al. 2017; Kagerbauer et al. 2019). On the other hand, while the methodological reliability of salivary oxytocin measures is controversial (Martins et al. 2020), evidence suggests that repeated measurements using radioimmunoassay (RIA) can enhance the reliability of salivary oxytocin assessment (Martins et al. 2020; Leng and Sabatier 2016; Martin et al. 2018; Tabak et al. 2023).

Existing evidence regarding oxytocin's circadian rhythm remains scarce and inconclusive. Van Dam et al. (2018) assessed salivary oxytocin in 12 adolescents at multiple times during the day and reported a sharp reduction immediately after awakening followed by stable levels during the day. Kagerbauer et al. (2019) measured oxytocin in CSF, blood, and saliva at four time points in 20 neurosurgical patients and reported circadian variations (decrease between early and late afternoon) only in CSF among postmenopausal women, but not in other participant subgroups or sampling methods. Both studies were well designed but limited by small sample sizes and heterogeneous participant groups, particularly regarding sex and hormonal status. These aspects are important since menstrual cycle phase and hormonal contraception influence oxytocin secretion due to its interactions with estrogen and progesterone (Quintana et al. 2024). Graugaard‐Jensen et al. (2017) addressed some of these limitations by comparing plasma oxytocin levels between naturally cycling women and women using oral contraceptives and found no significant circadian variation. However, morning samples were not collected immediately after individual awakening, as done for cortisol circadian assessment (Stalder et al. 2022).

In the present study, we aimed to investigate potential circadian variation in salivary oxytocin levels among young adult women. To address methodological limitations highlighted in previous research, we standardized saliva collection times, recruited a homogeneous sample regarding sex (female only) and age range, assessed all participants during the luteal phase of their menstrual cycle, and accounted for hormonal contraceptive use and antidepressant intake in clinical participants. By implementing these controls, we intended to reduce variability observed in earlier studies and contribute to a clearer understanding of oxytocin's circadian patterns.

We included both healthy adult women and women diagnosed with borderline personality disorder (BPD). It is of particular interest to study oxytocin in this clinical group because core features of BPD—such as severe emotional dysregulation, interpersonal difficulties, and heightened stress reactivity (American Psychiatric Association 2013; Meehan et al. 2018; Paris 2018), are related to processes modulated by oxytocin, including emotion regulation, stress response, and interpersonal relationships (Carter et al. 2024; Quintana and Guastella 2020; de Jong et al. 2015). Prior research on basal oxytocin levels in BPD shows inconsistent results, with some studies reporting lower plasma or serum oxytocin levels and reduced receptor expression compared to healthy controls (Bertsch et al. 2013; Carrasco et al. 2020; Ebert et al. 2018), while others found no significant group differences (Bomann et al. 2017; Bonfig et al. 2022). Additionally, to our knowledge, circadian patterns of oxytocin secretion in BPD have not been explored, with the exception of a pilot investigation conducted by our group (Aboulafia‐Brakha et al. 2023). Recently, Bocchio Chiavetto et al. (2025) reported lower morning plasma oxytocin levels in BPD, although the exact timing of this morning assessment was not specified. Therefore, the present study investigates circadian variation in salivary oxytocin levels in young adult healthy women and women diagnosed with BPD. By employing precise, timed sampling, it aims to further address existing gaps in the literature relevant to both groups.

## 2 Methods

The study was approved by the local ethics committee and was registered on ClinicalTrials.gov (NCT05357521). Recruitment and data collection are complete. The protocol included measures of oxytocin and cortisol under naturalistic conditions and during an experimental stress task. The present report focuses on the naturalistic measures of all participants, while results related to the experimental task will be presented separately since they are related to different research questions.

### 2.1 Participants

A hundred and twenty‐three (123) female participants (61 healthy controls and 62 patients with BPD) were included in the study and signed informed consent. Of these, 91 (47 healthy controls and 44 patients with BPD) completed the entire protocol. Healthy controls were recruited by advertisement and patients with BPD within patients treated at the Emotion Regulation Unit of the Division of Psychiatry, Geneva University Hospitals, as previously described (Blay et al. 2023).

Female participants aged 18–35 years were eligible for the study if they were free from neurohormonal or neurological disorders and not using systemic corticosteroids. Exclusion criteria for all participants included pregnancy or breastfeeding within the last 6 months, a history of alcohol or drug addiction, or a body mass index under 17. Participants experiencing major stressful life events (e.g., loss of a close relative, loss of employment, significant changes in marital status or medical condition) within the 2 months prior to enrolment were excluded initially or contacted for eligibility reassessment after a few months. Alcohol, tobacco, and occasional cannabis use were permitted, but use was prohibited on sampling days and controlled through interviews, with sampling rescheduled if necessary.

For the BPD group, participants were required to meet DSM‐5 criteria, confirmed via structured clinical interview. They were excluded if they had a formal diagnosis of psychosis, were daily users of neuroleptics, or were using antidepressants outside of the SSRI or SNRI classes. Healthy control women were excluded if they met three or more BPD diagnostic criteria (the threshold for a formal diagnosis being five).

### 2.2 Study Procedures

The entire protocol comprised three visits.

- Visit 1: During their initial visit, participants provided informed consent, underwent eligibility screening, completed a structured clinical interview, and filled out psychometric questionnaires.
- Visit 2: Naturalistic data collection: This visit involved the collection of naturalistic data over a single weekday. Participants collected salivary samples at six predetermined time points: upon awakening, 30 min post‐awakening, 45 min post‐awakening, between 12:00 and 2 pm (early afternoon) in the laboratory, at 6 pm, and immediately before sleep. To enhance adherence, participants received a reminder the evening before the sampling day and were instructed to precisely record the time of each collection.
  For women not using hormonal contraception, Visits 2 and 3 were scheduled to coincide with the luteal phase of their menstrual cycle, ideally 1 week prior to the expected onset of menstruation. Participants tracked their cycles and reported their average length to allow for individual estimation of the mid‐cycle and calculation of the ideal luteal phase timeframe (6 days subtracted from the expected end of their cycle). If menstruation began before a scheduled Visit 2 or 3, data collection was rescheduled to maintain cycle consistency. The selection of the luteal phase was based on prior research examining salivary oxytocin and cortisol interactions (Alley et al. 2019; Bernhard et al. 2018) and the broader aim of facilitating future inclusion of male participants by focusing on a phase with more similar stress responses between sexes compared to the follicular phase (Kirschbaum et al. 1999).
- Visit 3: The third visit was scheduled for the day after Visit 2, or at most 4 days later. This visit included an experimental stress task (data not reported in this paper) and the return of the evening salivary samples collected during the naturalistic assessment day.

### 2.3 Hormone Sample Collection and Processing

Saliva samples were collected using Salivettes with a synthetic swab (Sarstedt, Germany), which participants were instructed to chew for at least 1 min. Following collection, swabs were temporarily stored at −20°C. For long‐term storage until analysis, samples were centrifuged and stored at −80°C. Sample temperature was maintained with dry ice during shipping.

### 2.4 Hormonal Measures

Salivary concentrations of oxytocin and cortisol were quantified from the collected samples.

#### 2.4.1 Oxytocin

Salivary oxytocin concentrations (expressed in pg/mL) were quantified by radioimmunoassay (RIAgnosis, Sinzing, Germany). Consistent with the planned protocol and considering the lack of well‐established validity for long‐term oxytocin stability at room temperature or with home refrigeration (relevant for evening samples returned at a later visit), oxytocin was measured only at the first morning time point (upon awakening) and the fourth time point in the early afternoon (collected between 12:00 and 2 pm in the laboratory). The saliva samples collected at these two time points were split into two vials each to allow for the measurement of both oxytocin and cortisol from the same biological material. Detailed procedures for oxytocin quantification followed those described in previous studies, including our own (de Jong et al. 2015; Aboulafia‐Brakha et al. 2023; Bernhard et al. 2018).

#### 2.4.2 Cortisol

Salivary cortisol concentrations (expressed in Concentration (μg/dL)) were quantified (FCBG, Geneva, Switzerland) at all six collection time points using a direct ELISA kit for human salivary cortisol from IBL Tecan with a mean intra‐assay variability of 4.3% and a mean inter‐assay variability of 13.2%.

### 2.5 Statistical Analysis

Statistical analyses were conducted using SPSS 28.0. Oxytocin levels were normally distributed at both time points (Shapiro–Wilk test, *p* > 0.05 for both time points) and no outliers were identified (SPSS 1.5 interquartile range rule). For oxytocin analyses, a mixed 2 (Group: females with BPD vs. Healthy female controls) × 2 (Time: Awakening, early afternoon) repeated‐measures ANOVA was performed to assess circadian variation in salivary oxytocin levels and group differences across time. Effect sizes were reported using partial eta squared (ηp^2^). Box's test of equality of covariance matrices indicated that the assumption of homogeneity was met (*p* > 0.05). To explore the potential influence of hormonal contraception, a repeated‐measures ANCOVA was conducted, including contraceptive use as a covariate. Given that antidepressant use was exclusive to the BPD group, a separate repeated‐measures ANOVA was conducted within this group to test whether antidepressant use influenced circadian changes in salivary oxytocin. All statistical tests were two‐tailed, with a significance level set at *p* < 0.01.

For cortisol analyses, a mixed 2 (Group: females with BPD vs. Healthy female controls) × 6 (Time: Awakening, 30 min post‐awakening, 45 min post‐awakening, early afternoon, 6 pm and Bedtime) repeated‐measures ANOVA was performed.

## 3 Results

### 3.1 Sample Characteristics

As shown in Tables 1 and 2, groups did not significantly differ regarding age, body mass index (BMI), use of hormonal contraception, or cycle length.

**TABLE 1:** Group characteristics.

	Females with BPD (*n* = 44)	Healthy female controls (*n* = 47)	Test statistic	*p*
Age (years)	24.4 ± 5.07 (18–35)	25.1 ± 4.38 (18–35)	*U* = 767 (Mann–Whitney)	0.46
Body mass index	24.0 ± 5.35 (17.5–46.9)	22.2 ± 3.74 (18.0–41.7)	*U* = 646 (Mann–Whitney)	0.06
Hormonal contraception	12 (27.3%)	15 (31.9%)	*χ* ^2^(1) = 0.49	0.63

*Note:* M ± SD (range).

Abbreviation: BPD, Borderline personality disorder.

**TABLE 2:** Cycle duration in natural cycling women.

Cycle duration (days)	Females with BPD (*n* = 32)	Healthy female controls (*n* = 32)
< 26	3	4
27–30	18	19
30–35	9	8
> 35	2	1

### 3.2 Oxytocin

**FIGURE 1:**
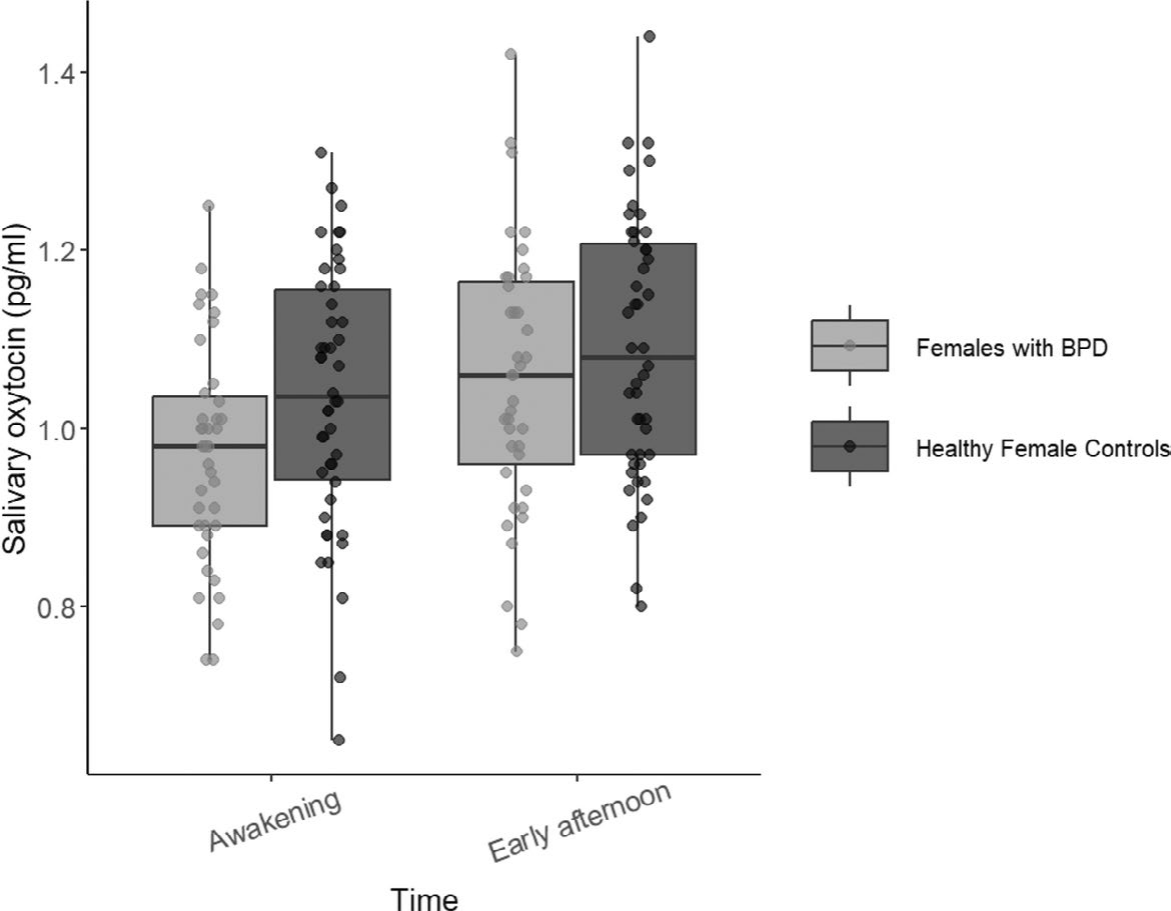
Salivary oxytocin at awakening and in the early afternoon in females with borderline personality disorder (BPD) and healthy female controls.

Figure 1 shows salivary oxytocin levels (pg/mL) at awakening and early afternoon for females with BPD and healthy female controls. The repeated‐measures ANOVA revealed a significant main effect of Time (*F*(1,83) = 28.03, *p* < 0.001, η^2^p = 0.25), indicating that salivary oxytocin levels significantly differed between awakening and early afternoon. There was no significant main effect of Group (*F*(1,83) = 3.23, *p* = 0.08), nor significant Group × Time interaction (*F*(1,83) = 1.17, *p* = 0.28). As salivary oxytocin was missing at one of the two time points for one healthy female and five females with BPD (due to low amounts of saliva after centrifugation), we imputed the missing values with the corresponding available time point value (a conservative approach) and ran the ANOVA with these imputed values. Effects of time remained significant (*F*(1, 89) = 26.80, *p* < 0.001), with no group effects or Group × Time interaction.

A total of 27 women in the entire sample (15 healthy female controls and 12 patients with BPD) reported the use of hormonal contraception (see Table 3 for a detailed description of contraception type). When hormonal contraception was included as a covariate, the main effect of Time remained significant (*F*(1,82) = 14.5, *p* < 0.01), with no significant main effect of group or Group × Time interaction.

**TABLE 3:** Hormonal contraception: Type and administration method.

	Females with BPD	Healthy female controls
Progestin only	2 subcutaneous implants 3 oral pill 3 intrauterine device	3 subcutaneous implants 8 oral pill
Combined progestin and estrogen	1 skin patch 3 oral pill	1 skin patch 3 oral pill
	Total = 12	Total = 15

Among women with BDP, 11 were treated with an SSRI antidepressant. A repeated‐measures ANOVA showed a significant main effect of time (*F*(1, 37) = 17.84, *p* < 0.001), but no main effect of group (*F*(1, 37) = 0.85, *p* = 0.36) or Time × Antidepressant interaction (*F*(1, 37) = 1.15, *p* = 0.29).

### 3.3 Cortisol

**FIGURE 2:**
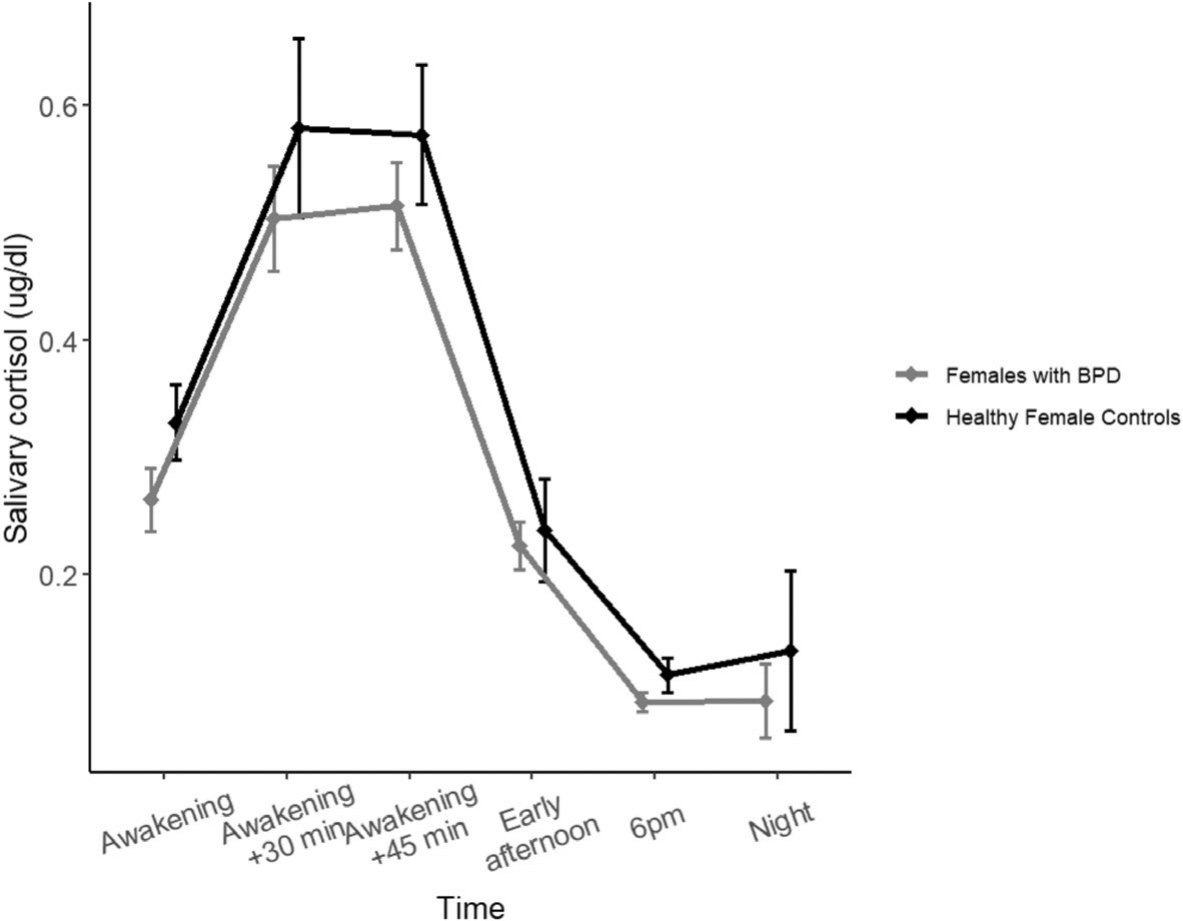
Salivary cortisol across time points in females with borderline personality disorder (BPD) and healthy female controls.

Figure 2 shows salivary cortisol levels across time points for females with BPD and healthy female controls. The repeated‐measures ANOVA revealed a significant main effect of Time (*F*(5,78) = 44.21, *p* < 0.001, η^2^p = 0.36), indicating that salivary cortisol levels significantly varied across time. There was no significant main effect of Group (*F*(1,78) = 2.09, *p* = 0.15), nor significant Group × Time interaction (*F*(5,78) = 0.25, *p* = 0.96). When missing values were imputed (eight at awakening, two at mid‐day and 1 at 6 pm), the effect of time remained significant, with no main effect of group or interaction.

## 4 Discussion

This study investigated potential circadian variation in salivary oxytocin levels among young adult women, including patients with BPD and healthy controls. Across the entire sample, we observed a significant increase in salivary oxytocin from awakening to early afternoon. While there were no significant group differences or interactions, we observed a nonsignificant trend (*p* = 0.08) toward lower oxytocin levels in the BPD group. Overall findings indicate the importance of considering the time of day in studies utilizing salivary oxytocin as a measure. The subsequent discussion will interpret these results within the context of existing literature and explore their implications for understanding oxytocin dynamics.

Previous studies investigating the diurnal variation of oxytocin levels showed inconsistent findings. Some studies indicated circadian patterns, such as a decline at awakening with stable daytime levels (Van Dam et al. 2018) or changes observed from morning to afternoon in specific populations (e.g., postmenopausal women) (Kagerbauer et al. 2019). In contrast, the absence of significant diurnal oxytocin variation was also reported (Graugaard‐Jensen et al. 2017). The differences between our findings and those of prior studies may be related to methodological variations. Our study included a larger and relatively homogeneous sample of young adult women and controlled for factors such as menstrual cycle phase, hormonal contraception, and medication use. Furthermore, the specific time points for sample collection in our protocol (awakening and early afternoon) differed from some previous studies. Another distinction is that our study utilized a nonhospitalized sample assessed within participants' natural environment, whereas some previous studies employed inpatient protocols (Kagerbauer et al. 2019). In contrast, the absence of significant diurnal oxytocin variation was also reported (Graugaard‐Jensen et al. 2017). The differences between our findings and those of prior studies may be related to methodological variations. Our study included a larger and relatively homogeneous sample of young adult women and controlled for factors such as menstrual cycle phase, hormonal contraception, and medication use. Furthermore, the specific time points for sample collection in our protocol (awakening and early afternoon) differed from some previous studies. Another distinction is that our study utilized a nonhospitalized sample assessed within participants' natural environment, whereas some previous studies employed inpatient protocols (Van Dam et al. 2018; Graugaard‐Jensen et al. 2017; Kagerbauer et al. 2019). This approach may have helped to minimize potential confounds associated with the stress of a hospital setting or concurrent medical interventions, factors that can influence oxytocin release (de Jong et al. 2015; Alley et al. 2019; Bernhard et al. 2018; Engert et al. 2016).

Beyond the observed circadian pattern, we noted a nonsignificant trend toward lower salivary oxytocin levels in the BPD group compared to healthy controls (*p* = 0.08). This observation presents a point of interest when considered alongside previous studies that have reported lower plasma oxytocin levels under basal conditions in individuals with BPD (Bertsch et al. 2013; Carrasco et al. 2020; Ebert et al. 2018; Bocchio Chiavetto et al. 2025). Direct comparisons are challenging due to differences in the type of measurement (saliva vs. plasma or receptor expression) and potential variations in the clinical characteristics of the BPD samples, as well as methodological factors in sample collection and analysis. While our finding did not reach statistical significance, it aligns with the direction of effects observed in some previous plasma‐based studies, suggesting this warrants further investigation in larger salivary oxytocin studies in this population.

Within the BPD group, a subset of participants was treated with antidepressant medication, restricted to Selective Serotonin Reuptake Inhibitors (SSRIs). Subgroup analyses comparing BPD participants on SSRIs versus those not on SSRIs did not reveal significant differences in salivary oxytocin circadian variation or overall levels. However, it is important to acknowledge the inherent heterogeneity within the BPD diagnosis, which encompasses a wide range of clinical profiles, symptom severity, and comorbidity. Given this variability, a more detailed characterization of clinical profiles in future studies could provide valuable insights into potential associations with oxytocin dynamics. Furthermore, exploring the effects of other classes of psychotropic medications (not included in our current study) would merit investigation.

In addition to examining group differences, we accounted for potential confounding variables known to influence oxytocin levels (Audunsdottir and Quintana 2022; Quintana et al. 2024). The groups did not significantly differ with respect to age, menstrual cycle phase, or the use of hormonal contraception. While hormonal contraception was included as a covariate in our analyses, we did not observe a significant effect of hormonal contraception on the circadian variation of salivary oxytocin, nor a significant interaction with group. However, it is important to note that previous research suggests that hormonal contraception can influence oxytocin release (Quintana et al. 2024; Garforth et al. 2020). Given these findings and the potential for hormonal fluctuations to impact oxytocin dynamics, continued assessment and control for variables such as age, menstrual cycle phase, and hormonal contraception use remain important considerations in future studies investigating oxytocin.

This study presents certain limitations that warrant consideration. Foremost, the sample was restricted to young adult women aged 18–35 years during the luteal phase of the menstrual cycle. Consequently, our findings may not be generalizable to males, women in other menstrual cycle phases, or premenarchal or postmenopausal individuals. While the luteal phase was selected for data collection to facilitate comparison with previous research examining oxytocin and cortisol interactions (Alley et al. 2019; Bernhard et al. 2018), we recognize that this phase is characterized by higher fluctuations in sex hormones, which may introduce variability (Quintana et al. 2024; Schmalenberger et al. 2021). This contrasts with some studies that have focused on assessing oxytocin during the more stable follicular phase [e.g., Jobst et al.]. Furthermore, our estimation of menstrual cycle phase was based on self‐report, and we lacked direct hormonal measures of estrogen and progesterone. Given the known interactions between sex hormones and oxytocin (Quintana et al. 2024; Schmalenberger et al. 2021), future studies including objective hormonal profiling would provide a more accurate understanding. Additionally, although the current sample size was sufficient to detect within‐subject circadian variation, future protocols designed to comprehensively assess circadian oxytocin dynamics could benefit from including a greater number of sampling time points throughout the day, potentially extending to later assessments, similar to standard cortisol protocols. However, this approach faces challenges related to the stability of salivary oxytocin without appropriate refrigeration, particularly for evening samples in outpatient contexts.

Despite these limitations, our findings yield important methodological and theoretical implications. The presence of circadian variation in salivary oxytocin levels in young adult women underscores the necessity for future studies to carefully control for the time of day at which samples are collected. Implementing such standardized collection protocols can reduce methodological variability and thereby enhance the replicability and interpretability of oxytocin research findings. Conversely, a failure to account for these diurnal fluctuations may contribute to inconsistencies observed across studies.

## Author Contributions

**M. Teixeira de Almeida:** investigation, writing – original draft, data curation, formal analysis. **L. Quattrocchi:** investigation, writing – original draft, writing – review and editing, formal analysis. **N. Perroud:** funding acquisition, resources, supervision, writing – original draft. **T. Aboulafia‐Brakha:** conceptualization, methodology, formal analysis, project administration, supervision, data curation, writing – review and editing, writing – original draft, validation, funding acquisition.

## Ethics Statement

Local ethical committee approval (Commission Cantonale d'éthique de la recherche Genèvel): Swissethics NAC 2022‐00067.

## Conflicts of Interest

The authors declare no conflicts of interest.

## Data Availability

The data that support the findings of this study are available from the corresponding author upon reasonable request.
